# Speciation and sorption of phosphorus in agricultural soil profiles of redoximorphic character

**DOI:** 10.1007/s10653-020-00561-y

**Published:** 2020-04-23

**Authors:** Karen Baumann, Sabry M. Shaheen, Yongfeng Hu, Peter Gros, Elena Heilmann, Mohsen Morshedizad, Jianxu Wang, Shan-Li Wang, Jörg Rinklebe, Peter Leinweber

**Affiliations:** 1 Soil Sciences, Faculty for Agriculture and Environmental Sciences, University of Rostock, Justus-von-Liebig-Weg 6, 18051 Rostock, Germany; 2 Laboratory of Soil- and Groundwater-Management, School of Architecture and Civil Engineering, Institute of Foundation Engineering, Water- and Waste-Management, University of Wuppertal, Pauluskirchstraße 7, 42285 Wuppertal, Germany; 3 Department of Soil and Water Sciences, Faculty of Agriculture, University of Kafrelsheikh, Kafr El-Sheikh, 33516 Egypt; 4 Canadian Light Source, University of Saskatchewan, Saskatoon, SK S7N 2V3 Canada; 5 State Key Laboratory of Environmental Geochemistry, Institute of Geochemistry, Chinese Academy of Sciences, Guiyang, 550082 People’s Republic of China; 6 Department of Agricultural Chemistry, National Taiwan University, 1 Sect. 4, Roosevelt Rd., Taipei, 10617 Taiwan; 7 Department of Environment, Energy and Geoinformatics, University of Sejong, 98 Gunja-Dong, Guangjin-Gu, Seoul, Republic of Korea

**Keywords:** Controlled drainage, Adsorption isotherm, P mobilization, Redox, Synchrotron, XANES

## Abstract

Controlled drainage is considered as a soil management tool to improve water supply to crops and reduce nutrient losses from fields; however, its closure may affect phosphorus (P) mobilization in soil. To assess the P mobilization potential, three soil profiles with redoximorphic features were selected along a slight hill in Northern Germany. Soil samples from three depths of each profile were characterized for basic properties, total element content, oxalate- and dithionite-extractable pedogenic Al, Fe and Mn (hydr)oxides, P pools (sequential extraction), P species [P *K*-edge X-ray absorption near-edge structure (XANES) spectroscopy] and P sorption behavior. In topsoil (~ 10 cm depth), labile P (H_2_O-P + resin-P + NaHCO_3_-P) accounted for 26–32% of total P (P_t_). Phosphorus *K*-edge XANES revealed that up to 49% of P_t_ was bound to Al and/or Fe (hydr)oxides, but sequential fractionation indicated that > 30% of this P was occluded within sesquioxide aggregates. A low binding capacity for P was demonstrated by P sorption capacity and low *K*_f_ coefficients (20–33 ${\text{mg}}^{{1 - n_{\text{f}} }} \,{\text{L}}^{{n_{\text{f}} }} \,{\text{kg}}^{ - 1}$) of the Freundlich equation. In the subsoil layers (~ 30 and ~ 65 cm depth), higher proportions of Al- and Fe-bound P along with other characteristics suggested that all profiles might be prone to P mobilization/leaching risk under reducing conditions even if the degree of P saturation (DPS) of a profile under oxic conditions was < 25%. The results suggest that a closure of the controlled drainage may pose a risk of increased P mobilization, but this needs to be compared with the risk of uncontrolled drainage and P losses to avoid P leaching into the aquatic ecosystem.

**Electronic supplementary material:**

The online version of this article (10.1007/s10653-020-00561-y) contains supplementary material, which is available to authorized users.

## Introduction

In Germany, draining wet and waterlogged land for the establishment of agriculturally used fields is a common practice. In northern Germany, however, climate change results in the decrease of precipitation during growing season but also extreme precipitation events (Madsen et al. 2014; Svoboda et al. 2015). To mitigate these extremes, controlled drainage is considered as an opportunity not only to retain moisture in fields for improving crop performance (Tolomino and Borin 2019) but also to control diffuse phosphorus (P) losses from fields at watershed scale (Carstensen et al. 2019; Dagnew et al. 2019). An increased water table can lead to reductive conditions, which are likely to mobilize P that was previously held by pedogenic oxides as shown for peat soils (Meissner et al. 2008; Herndon et al. 2019). Also, Valero et al. (2007) reported an increase in P mobilization in a Canadian Humic Gleysol at increased water table. Other studies, however, detected decreased P loads in controlled drainage water (Wesström and Messing 2007; Jouni et al. 2018).

In soil, P generally is chemically bound within minerals (e.g., apatite) and organic substances (e.g., phospholipids), is sorbed to the minerals or organic surfaces predominantly as phosphate ion and occurs in the equilibrium soil solution as H_2_PO_4_^−^ or HPO_4_^2−^. Under oxic conditions, phosphate ions might be sorbed to binding sites of clay minerals and/or Al, Fe and Mn (hydr)oxides with which they can form insoluble complexes (Lijklema 1980; Penn et al. 2005; Johnson et al. 2016). However, if controlled drains are closed, increased water content may lead to reducing conditions in soil. In this case, microbial respiration leads to reductive dissolution of Mn(III, IV) and Fe(III) (hydr)oxides, which enhances P availability in the soil solution by the release of P adsorbed to minerals (Lovley 1991; Peretyazhko and Sposito 2005; Maranguit et al. 2017). This mechanism of phosphate mobilization has been successfully employed by flooding of soils to increase the P availability and thus the P nutritional status of, e.g., rice plants (Islam and Islam 1973; Seng et al. 1999; Rakotoson et al. 2015). However, enhanced P mobilization may also be of disadvantage since increased P solubility can lead to P leaching and nutrient loss with its unfavorable consequences not only for crop plants but also for aquatic ecosystems (Sims et al. 1998; Nausch et al. 2017). King et al. (2015) reported that subsurface tile drainage accounted for nearly 50% of dissolved P exported at the watershed scale in central Ohio, USA. At the watershed scale, various factors influencing P sorption, bioavailability or leachability are integrated in texture (Leinweber et al. 1999), mineral composition (Gérard 2016), pH (Shaheen et al. 2009; Penn and Camberato 2019), soil organic matter (SOM) content (Shaheen and Tsadilas 2013; Yang et al. 2019) and also the oxidation status of a soil (Pant and Reddy 2001) and interact in fields under controlled drainage.

An estimation of potential P availability has often been based on total soil P concentrations, P lability, the presence of P binding sites and/or has been characterized by P adsorption behavior of the soil simulated by adsorption isotherms in batch equilibrium experiments (Leinweber et al. 1999; Maguire et al. 2001; Shaheen et al. 2007, 2009; Braun et al. 2019). Some studies also used P species determined by P *K*-edge X-ray absorption near-edge structure (XANES) spectroscopy to estimate P availability under different fertilization scenarios (Eriksson et al. 2016; Koch et al. 2018). This method is based on measuring the variation of the absorption coefficient of a sample induced by different X-ray energies and is sensitive to the P oxidation state and local environment of a P atom (e.g., bond angle, geometry of coordinating cations) (Calvin 2013). Recently, Ippolito et al. (2019) showed different P availability under different irrigation systems based on P *K*-edge XANES results, which implies that this technique may reveal helpful indications in terms of P availability assessment for fields under controlled drainage.

Phosphorus availability in soil is often assessed from top soil samples only (Leinweber et al. 1997; Kleinman et al. 2000; Maguire et al. 2001; Tóth et al. 2013; Jarosch et al. 2018). However, deeper soil layers may additionally contribute positively to P availability (Koch et al. 2018) or may provide further binding sites for P which could reduce P availability and thus P leaching (Djodjic et al. 2004). A thorough characterization of top and deeper soil layers regarding potential P availability and mobilization risk is necessary to provide the base for redox-caused P release scenarios as they may occur in fields under controlled drainage. Thereby, also the hill slope position of a soil profile may most likely be important since erosion processes could not just have affected P contents but also P binding sites, which in turn impact potential P availability and leaching risk.

Phosphorus leaching reduction is of particular interest for arable land in Mecklenburg-Western Pomerania (northeastern Germany) since catchments drain into the Baltic Sea for which a reduction in inputs is targeted by the Helsinki commission (HELCOM) convention (HELCOM 2007). The current study therefore aimed to assess the P situation in three soil profiles along a catena in this region under installed drainage to estimate possible effects of controlled drainage on P availability/mobilization risk in these soils. Three different soil depths were considered by investigating P speciation, fractionation and adsorption isotherms of P as affected by soil basic characteristics and pedogenic oxides content. Additionally, this study clarifies whether P *K*-edge XANES analyses of bulk soil samples run at two different synchrotrons yield similar results.

## Materials and methods

### Soil sampling

In May 2018, soil samples were taken from an experimental field at Dummerstorf (Mecklenburg-Western Pomerania, Germany), which had been used for studies of diffuse P losses (Tiemeyer et al. 2009), and was cropped by a grass–lucerne–mixture for fodder at the time of sampling. In the course of soil monolith sampling (for lysimeter experiments), three soil profiles had been excavated by drilling at the upper slope (upper slope), at mid-slope position (mid-slope) and at the bottom of a slight slope (toe slope) (Table 1). At each site, four replicates, each being a mixture of soil from two 250-ml soil cores, were sampled from three depths (1, 2, 3; Table 1). In the following, a specific site (“upper slope,” “mid-slope,” “toe slope”) and depth (“1,” “2,” “3”) are referred to as, e.g., “upper slope-1,” meaning depth 1 of the upper-slope profile. Soil samples were air-dried or dried at 105 °C, sieved to < 2 mm and partly finely ground before further analyses.

**Table 1:** Geographical position, soil classification, soil horizons and soil sampling depths of three soil profiles along a slope in Dummerstorf, Germany

	Upper slope	Mid-slope	Toe slope
Geographical position (WGS 84)			
N	54.005280°	54.004112°	54.003261°
E	12.252500°	12.252461°	12.252568°
m a.s.l.	42	41	39
Soil classification			
WRB (IUSS Working Group 2014)	Stagnic Cambisol (stCM)	Haplic Stagnosol (haST)	Colluvic Stagnosol (coST)
German Soil Classification System (AG Boden 2005)			
Soil type	Braunerde-Haftpseudogley (BB-SH)	Normpseudogley (SSn)	Pseudogley-Kolluvisol (SS-YK)
Substrate type	Moraine sand over moraine loam	Moraine sand over moraine loam	Moraine sand over moraine loam
	(g-s/g-l)	(g-s/g-l)	(g-s/g-l)
Soil profile (horizon, depth)			
	**Ap (0–27** **cm)**	**Ap (0–30** **cm)**	**Ap (0–28** **cm)**
	**Bv (27–37** **cm)**	**Sw (30–60** **cm)**	**M (28–43** **cm)**
	**Sg-Bv (37–87** **cm)**	**Sd (60–90** **cm)**	eSw (43–55 cm)
	ilCv (87–90 cm)		**eSd (55–90** **cm)**
Soil sampling depth			
Depth 1	7–14 cm	7–14 cm	7–14 cm
Depth 2	27–34 cm	32–39 cm	32–39 cm
Depth 3	55–62 cm	65–72 cm	65–72 cm

Sampled horizons are marked in bold

### Soil texture, pH and total element contents

Soil texture (sieving and sedimentation procedure), bulk density (mass of 105 °C dry soil per cm^3^) and pH (0.01 M CaCl_2_, 1:2.5 w:v; pH meter pH540 GLP WTW) were determined by standard procedures on soil < 2 mm (Blume et al., 2011; ISO 11272:^2017^). Pore volume was estimated as described in Wessolek et al. (2009).

The contents of total C (C_t_), N and S were obtained by dry combustion of finely ground soil samples using an elemental analyzer (VARIO EL, Elementar Analysensysteme GmbH, Hanau, Germany). Inorganic C (C_inorg_) content was determined by a Scheibler calcimeter, and organic C (C_org_) content was calculated by subtracting C_inorg_ from C_t_ content (Blume et al. 2011).

Total Al, Ca, Fe, K, Mg, Mn and P were extracted from 0.5 g finely ground soil samples by microwave-assisted digestion with aqua regia solution (3:1 hydrochloric acid/nitric acid) (Chen and Ma 2001), and their concentrations were subsequently determined by inductively coupled plasma optical emission spectroscopy (ICP-OES, Perkin-Elmer Optima 8300 DV, Waltham, MA, USA).

### Extraction of pedogenic oxides

The Al, Fe, Mn and P from poorly crystalline pedogenic oxides were extracted from 0.5 g soil (< 2 mm) using 0.2 M NH_4_ oxalate solution (pH 3) in the dark (Schwertmann 1964). The total amount of Fe, Al, Mn from pedogenic oxides (poorly and well-crystallized oxides) was extracted by the dithionite–citrate–bicarbonate (DCB) method after Mehra and Jackson (1960) with slight modifications. In brief, 1 g of soil (< 2 mm) was incinerated at 550 °C before twice extracted with 25 ml citrate–bicarbonate solution and 1 g Na dithionite at 80 °C and washed with 10 ml 0.1 M MgSO_4_ solution thereafter. Concentrations of elements in the oxalate extract (Fe_ox_, Al_ox_, Mn_ox_, P_ox_) and DCB extract (Fe_dit_, Al_dit_, Mn_dit_) were measured by ICP-OES (Perkin-Elmer Optima 8300 DV, Waltham, MA, USA). The amount of pedogenic oxides with a higher degree of crystallinity was estimated by subtracting the amount of poorly crystalline oxides from the amount of total pedogenic oxides (Al_dit-ox_, Fe_dit-ox_, Mn_dit-ox_).

Phosphorus sorption capacity (PSC) and the degree of P saturation (DPS) of a soil were determined after Maguire et al. (2001) including Mn using Eqs. 1 and 2, respectively.

1 $${\text{PSC}} = \alpha \cdot \left( {{\text{Al}}_{\text{ox}} + {\text{Fe}}_{\text{ox}} + {\text{Mn}}_{\text{ox}} } \right)$$

where PSC is the P sorption capacity (mmol kg^−1^), α is the applied scaling factor of 0.5 and Al_ox_ + Fe_ox_ + Mn_ox_ is the sum of oxalate-extractable Al, Fe and Mn (mmol kg^−1^).

2 $${\text{DPS}} = \frac{{{\text{P}}_{\text{ox}} }}{\text{PSC}} \cdot 100\%$$

where DPS is the degree of P saturation (%), P_ox_ is the amount of oxalate-extractable P (mmol kg^−1^) and PCS is the P sorption capacity (mmol kg^−1^).

The weighted mean DPS for a profile (up to 87 cm depth) was calculated by Eq. (3).

3 $${\text{DPS}}_{\text{profile}} = \frac{{h_{{{\text{depth}}\, 1}} \cdot {\text{DPS}}_{{{\text{depth}}\, 1}} + h_{{{\text{depth}}\, 2}} \cdot {\text{DPS}}_{{{\text{depth}}\, 2}} + h_{{{\text{depth}}\, 3}} \cdot {\text{DPS}}_{{{\text{depth}}\, 3}} }}{{87\, {\text{cm}}}}$$

where DPS_profile_ is the degree of P saturation of the profile up to 87 cm depth (%), soil horizon thickness, *h*, is the thickness of a soil layer (cm) and DPS_depth_ is the degree of P saturation at a certain soil depth (%).

### Sequential P fractionation

Sequential P fractionation was conducted after Hedley et al. (1982) with slight modifications. In brief, 0.5 g finely ground soil was extracted sequentially by double-distilled water (50 ml) (H_2_O-P), double-distilled water in the presence of anion exchange resin (30 ml; 6 × 2 cm resin membrane; 55164 2S, BDH Laboratory Supplies, Poole, England) (resin-P), 0.5 M NaHCO_3_ at pH 8.5 (50 ml) (NaHCO_3_-P), 0.1 M NaOH (50 ml) (NaOH-P) and 1 M H_2_SO_4_ (30 ml) (H_2_SO_4_-P). Residual P was calculated as the difference between P_t_ extracted by aqua regia solution and the sum of total P of the various fractions. The concentration of total P in the extracts was determined by ICP-OES at 214-nm wavelength. The sum of H_2_O-P, resin-P and NaHCO_3_-P was considered as labile P, whereas NaOH-P was assigned to moderately labile P (Fe- and Al-associated P), H_2_SO_4_-P to relatively stable Ca-P compounds and residual-P to not extractable P (Tiessen and Moir 1993).

### P *K*-edge XANES spectroscopy

The P *K*-edge XANES spectra were recorded at the Canadian Light Source (CLS) in Saskatoon, Saskatchewan, Canada, at the soft X-ray micro-characterization beamline (SXRMB) (Hu et al. 2010) and at the Taiwanese Light Source (TLS) at the National Synchrotron Radiation Research Center (NSRRC) in Hsinchu, Taiwan, at beamline 16A of the electron storage ring (1.5 GeV; bending magnet; beam current 361 mA). At the two beamlines, sample preparation was done according to each beamline standard procedure, and data acquisition was performed to gain spectra of highest possible quality (Table S1). For data processing (spectra averaging, background correction, normalization) and linear combination fitting (LCF), ATHENA software package (Demeter 0.9.25) was used (Ravel and Newville 2005). The LCF was performed in the energy range between -10 and +30 eV of E_0_. It was carried out for all possible binary to quaternary combinations using the following 12 P reference standards: for organic P (P_o_): C_6_H_18_O_24_P_6_·xNa^+^·yH_2_O (= phytic acid sodium salt hydrate) and lecithin, for Ca-P: CaHPO_4_·2H_2_O, and Ca_10_(PO_4_)_6_(OH)_2_ (= hydroxyapatite), for Mn-P: P-Mn (= P adsorbed to natural Mn concretion), for Fe-P: FePO_4_·2H_2_O, FePO_4_·4H_2_O, Fe_3_^2+^(PO_4_)_2_·8H_2_O (= vivianite), P-FeOOH (= P adsorbed on goethite) and P-Fe_2_O_3_ (= P adsorbed on ferrihydrite) and for Al-P: P-(Al(OH)_3_) (= P adsorbed on gibbsite) and P-AlOOH (= P adsorbed on boehmite). Only fits in which the share of each compound was ≥ 5% were used. The R-factors were used as goodness-of-fit criteria, and the significance between fits was evaluated using the Hamilton test (Calvin 2013) as described in Baumann et al. (2017). In case of similar probability of more than one fit (Hamilton *P* ≤ 0.05), the fit resulting from a lower number of combinations was given priority before fit proportions were averaged (Baumann et al. 2017).

### P adsorption isotherms

For sorption experiments, 1 g of soil (< 2 mm) was equilibrated in 25 ml 0.01 M CaCl_2_ solution of varying KH_2_PO_4_ concentrations (0, 0.5, 1, 1.5, 2, 3.5, 5, 10, 15, 20, 30, 50 mg P L^−1^). Samples were shaken end-over-end for 24 h at 20 rotations min^−1^ before they were centrifuged at 4500×*g* for 15 min. Phosphorus in the supernatant was quantified by ICP-OES. The amount of adsorbed P (*Q*_ads_) was calculated by Eq. 4 before the isotherm model after Freundlich (Eq. 5) or Langmuir (Eq. 6) was applied to simulate P adsorption to the soils. Phosphorus adsorption isotherm coefficients (*K*_L_*, K*_f_*, n*_f_) and maximum absorbable P concentration (*q*_L,max_) were estimated using the linearized form of the isotherm. Measurements were conducted in triplicate on one soil field replicate.

4 $$Q_{\text{ads}} = \frac{{C_{0} - C_{\text{eq}} }}{{V_{\text{eq}} \cdot m_{\text{soil}} }}$$

where *Q*_ads_ is the mass of adsorbed P (mg kg^−1^), *C*_0_ is the initial concentration of P (mg L^−1^) added, *C*_eq_ is the concentration of the equilibrated solution (mg L^−1^), *V*_eq_ is the volume of the equilibrium solution (L) and *m*_soil_ is the mass of soil (kg).

5 $$Q_{\text{ads}} = K_{f} \cdot C_{\text{eq}}^{{n_{f} }}$$

where *Q*_ads_ is the mass of adsorbed P (mg kg^−1^), *K*_f_ is the Freundlich unit capacity (${\text{mg}}^{{1 - n_{\text{f}} }} \,{\text{L}}^{{n_{\text{f}} }} \,{\text{kg}}^{ - 1}$), *C*_eq_ is the concentration of P in the equilibrated solution (mg L^−1^), and *n*_f_ is the Freundlich exponent describing the nonlinearity of the adsorption.

6 $$Q_{\text{ads}} = q_{{L , {\text{max}}}} \cdot \frac{{K_{L} \cdot C_{\text{eq}} }}{{1 + K_{L} \cdot C_{\text{eq}} }}$$

where *Q*_ads_ is the mass of adsorbed P (mg kg^−1^), *q*_L,max_ is the maximum adsorbed P concentration to cover the surface with a monolayer of P containing molecules (mg kg^−1^), *K*_L_ is the unit capacity (L mg^−1^) and *C*_eq_ is the concentration of P in the equilibrated solution (mg L^−1^).

### Statistics

A heteroscedastic *t* test for dependent and independent samples was used to test significant differences within soil profiles (depth) and between sites (hill position), respectively. Pearson’s correlation coefficient was calculated to reveal relationships between parameters. Statistical calculations were done using R software (version 3.5.3, R Development Core Team 2019). Unless stated otherwise, significant differences refer to *P* ≤ 0.05.

## Results

### Soil texture, pore volume and pH

The sequence of the soil horizons differed for the three profiles and was typical for a stagnic cambisol (Braunerde-Haftpseudogley) at the upper-slope position, a haplic stagnosol (Normpseudogley) at mid-slope position and a colluvic stagnosol (Pseudogley-Kolluvisol) at the toe-slope position according to the World Reference Base for Soil Resources (WRB) (IUSS Working Group WRB 2014) and German Soil Classification System (KA5; AG Boden 2005), respectively (Table 1). All soils showed redoximorphic affectation which could be deduced from distinct horizons of redoximorphic character (Sw, Sg). Soil texture was similar between all depths and profiles resulting in the texture class sandy loam according to the WRB (IUSS Working Group WRB 2014) (Table 2). According to the German KA5 (AG Boden 2005), the texture classes were Sl4 except for toe slope-2 and 3 which showed Sl3. Pore volume and bulk density ranged from 33 to 41% and 1.6 to 1.8 g cm^−3^, respectively. Pore volume was significantly higher at depth 1 than at depth 2 at the upper- and mid-slope profiles, whereas it was similar at depths 1 and 2 at the toe-slope profile. A higher pore volume (and lower bulk density) was determined for toe slope-2 compared with mid-slope-2. The pH values ranged from 6.52 to 6.88 and were similar throughout each profile except for the mid-slope-3, which had a slightly higher pH value compared to the other depths of this profile. The pH values were significantly lower at toe slope-1 and 2 compared with depths 1 and 2 from the mid-slope profile.

**Table 2:** Soil texture, pore volume, bulk density and pH in soils from three depths of the upper-, mid- and toe-slope soil profile

Hill position	Depth	Clay (g kg^−1^)	Silt (g kg^−1^)	Sand (g kg^−1^)	Pore volume (%)		Bulk density (g cm^−3^)		pH_CaCl2_	
Upper slope	1	142	311	547	39	bx	1.6	ax	6.64	axy
	2	145	297	557	35	axy	1.7	bxy	6.58	ax
	3	140	296	564	40	abx	1.6	abx	6.57	ax
Mid-slope	1	138	326	536	40	bx	1.6	ax	6.84	ay
	2	146	316	538	34	ax	1.7	by	6.86	ay
	3	148	273	579	34	abx	1.8	abx	6.88	by
Toe slope	1	130	350	520	41	bx	1.6	ax	6.50	ax
	2	119	364	517	38	by	1.6	ax	6.52	ax
	3	91	352	557	33	ax	1.8	bx	6.77	axy

Different letters indicate significant differences between depths within the same soil profile (a–c) and between profiles within the same depth (x–z), respectively; *n* = 4

### Total element contents

The total C content ranged between 1.5 and 15.3 g kg^−1^ (Table 3). Generally, C_t_ content was highest in depth 1 in the three profiles; however, C_t_ content in toe slope-1 was not significantly different from that in toe slope-3. In depth 1, C_t_ was higher in the toe-slope profiles compared with the upper-slope profile. Within the toe-slope profile, the C_inorg_ content ranged between 0.1 and 9.1 g kg^−1^ and was highest at depth 3. The C_inorg_ content was higher in mid-slope-1 compared with toe slope-1. The C_org_ content (ranging between 1 and 13.8 g kg^−1^) as well as N content (ranging between 0.2 and 1.3 g kg^−1^) decreased with increasing depth in all profiles and was higher at mid-slope-1 and toe slope-1 compared with upper slope-1. Total Ca content was highest at toe slope-3 (28.9 g kg^−1^). Highest Ca contents in depths 1 and 2 were found at the mid-slope profile, while the lowest Ca contents were at depths 1 and 2 of the upper-slope profile.

**Table 3:** Mean concentrations of total elements, inorganic C (C_inorg_) and organic C (C_org_) in soils from three depths of the upper-, mid- and toe-slope soil profile

Hill position	Depth	C_t_ (g kg^−1^)		C_inorg_ (g kg^−1^)		C_org_ (g kg^−1^)		N_t_ (g kg^−1^)		S_t_ (g kg^−1^)		Ca_t_ (g kg^−1^)		K_t_ (g kg^−1^)		Mg_t_ (g kg^−1^)		Al_t_ (g kg^−1^)		Fe_t_ (g kg^−1^)		Mn_t_ (mg kg^−1^)		P_t_ (mg kg^−1^)	
Upper slope	1	10.6	cx	0.54	axy	10.0	bx	1.0	cx	0.3	bx	1.9	ax	2.0	ax	2.0	ay	9.7	ax	12.1	ay	229	ax	470	bx
	2	7.0	bxy	0.08	ax	6.9	abxy	0.7	by	0.3	abxy	1.8	ax	2.0	aby	2.1	aby	10.3	ax	12.9	abz	230	ay	430	by
	3	1.5	ax	0.11	ax	1.4	axy	0.2	ax	0.2	ax	2.0	ax	2.7	by	2.8	by	11.7	ay	16.8	by	364	by	258	ax
Mid-slope	1	15.3	by	1.47	by	13.8	cy	1.3	cy	0.3	bx	7.6	bz	2.0	ax	2.2	az	8.8	ax	10.4	bx	222	bx	548	cy
	2	4.1	ay	0.12	ax	4.0	bx	0.3	bx	0.2	ax	2.7	az	1.6	axy	1.8	abx	11.5	ax	8.2	ay	111	ax	220	ax
	3	3.6	ax	1.55	abx	2.0	ay	0.2	ax	0.2	ax	5.9	abx	2.3	ay	2.7	bxy	12.3	ay	11.0	abx	119	ax	354	bx
Toe slope	1	13.5	by	0.14	ax	13.4	cy	1.3	cy	0.4	bx	2.7	by	1.8	bx	1.8	bx	8.6	bx	10.9	bx	448	by	557	by
	2	6.9	ax	0.13	ax	6.7	by	0.7	by	0.3	by	2.3	ay	1.3	ax	1.7	ax	8.7	bx	10.8	bx	363	az	302	ax
	3	9.9	aby	9.12	by	1.0	ax	0.1	ax	0.2	ax	28.9	cy	1.5	abx	1.9	cx	6.2	ax	8.0	ax	309	aby	322	ax

Different letters indicate significant differences in an element concentration between depths within the same soil profile (a–c) and between profiles within the same depth (x–z), respectively; *n* = 4

Total Al (Al_t_) content ranged between 6.2 and 12.3 g kg^−1^ and increased with depth in the upper- and mid-slope profile, while it was significantly lower at depth 3 of the toe-slope profile. Of all three profiles, the toe slope had the lowest Al_t_ content at depth 3, while the highest content was found in depth 3 of the mid-slope profile. Total Fe (Fe_t_) content ranged between 8.0 and 16.8 g kg^−1^. In the upper-slope profile, it increased with increasing depth, while in the toe-slope profile it decreased with increasing depth; on the other hand, in the mid-slope profile the lowest value was at depth 2, while the highest value was at depth 1. Total Mn (Mn_t_) content ranged between 111 and 448 mg kg^−1^. The toe-slope profile contained higher values of Mn_t_ than the upper- and mid-slope profiles. In the upper-slope profile, it was higher at depth 3 than at depths 2 and 1, while in the mid-slope and toe-slope profiles, it was higher at depth 1 than at depths 2 and 3. Total P (P_t_) content ranged between 220 mg kg^−1^in depth 2 of the mid-slope profile and 557 mg kg^−1^in depth 1 of the toe-slope profile. The top layer contained higher values of P_t_ than the sublayers in the three profiles.

### Pedogenic oxides, PSC and DPS

The content of highly crystallized Al_dit-ox_ ranged between 532 and 1385 mg kg^−1^ and accounted for 6–14% of Al_t_ with the highest concentrations in depth 1 of all soil profiles (Table 4). The Fe_dit-ox_ ranged between 1515 and 5277 mg kg^−1^ and accounted for 19–37% of Fe_t_. While the content of crystallized Fe_dit-ox_ was higher in depth 3 than in depths 1 and 2 in the upper-slope profile, it was higher in depth 1 in the mid-slope profile and higher in depth 2 in the toe-slope profile than in the other depths. The content of Mn_dit-ox_ ranged between 14 and 238 mg kg^−1^ and accounted for 5–54% of Mn_t_. It was higher in depth 1 than in depths 2 and 3 of the mid- and toe-slope profiles, while it was accumulated in depth 2 of the upper-slope profile.

**Table 4:** Mean concentrations of Al, Fe and Mn from pedogenic oxides and oxalate extractable P as well as P sorption capacity (PSC) and degree of P sorption (DPS) for soils from three depths of the upper-, mid- and toe-slope soil profile

Hill position	Depth	Al_dit-ox_			Fe_dit-ox_			Mn_dit-ox_			Al_ox_			Fe_ox_			Mn_ox_			P_ox_			PSC Al + Fe + Mn		DPS Al + Fe + Mn	
		(mg kg^−1^)		(%)	(mg kg^−1^)		(%)	(mg kg^−1^)		(%)	(mg kg^−1^)		(%)	(mg kg^−1^)		(%)	(mg kg^−1^)		(%)	(mg kg^−1^)		(%)	(mmol kg^−1^)		(%)	
Upper slope	1	1385	bx	(14)	4119	ay	(34)	33	bx	(14)	606	ax	(6)	2160	ay	(18)	134	ax	(59)	290	bx	(62)	32	ay	30	bx
	2	1379	by	(14)	4520	aby	(35)	39	by	(17)	624	ax	(6)	2155	az	(17)	131	ax	(57)	278	by	(65)	32	az	28	by
	3	991	ax	(8)	5277	by	(32)	18	ax	(5)	513	ax	(4)	1897	az	(11)	247	abx	(68)	109	ax	(43)	29	ay	12	abx
Mid-slope	1	1118	bx	(13)	3567	bx	(34)	40	cx	(18)	621	bx	(7)	1627	bx	(16)	117	bx	(53)	300	cxy	(55)	27	cx	36	cy
	2	1011	ax	(9)	1515	ax	(19)	16	bx	(15)	804	cxy	(7)	794	ax	(10)	39	ax	(35)	90	ax	(40)	22	bx	13	ax
	3	780	abx	(6)	2633	abx	(25)	14	ax	(11)	471	ax	(4)	1098	ay	(10)	33	aby	(29)	152	by	(45)	19	ax	26	by
Toe slope	1	1193	bx	(14)	4092	abxy	(37)	238	bx	(54)	610	ax	(7)	1845	bx	(17)	242	by	(54)	324	by	(58)	30	bxy	35	bxy
	2	998	ax	(12)	4016	by	(37)	67	ay	(18)	750	by	(9)	1448	by	(13)	204	ay	(56)	179	axy	(58)	29	by	20	axy
	3	532	abx	(8)	2501	ax	(31)	68	abx	(26)	383	abx	(7)	475	ax	(6)	217	abxy	(66)	96	ax	(30)	13	ax	25	aby

Elemental concentrations indexed by “dit-ox” represent crystalline pedogenic oxides; elemental concentrations indexed by “ox” represent poorly crystalline pedogenic oxides. Proportions (% of total element concentration) are given in brackets. Different letters indicate significant differences in an element concentration between depths within the same soil profile (a–c) and between profiles within the same depth (x–z), respectively; *n* = 4

The content of poorly crystalline Al (Al_ox_) ranged between 383 and 804 mg kg^−1^ and accounted for 4–9% of Al_t_. In all profiles, it was higher at depth 2 than at depths 1 and 3. The Fe_ox_ content ranged between 475 and 2160 mg kg^−1^ and accounted for 6–18% of Fe_t_, and it was higher in depth 1 than in depths 2 and 3 in all profiles. The highest Fe_ox_ contents were determined in the upper-slope profile. The Mn_ox_ content ranged between 33 and 247 mg kg^−1^ and accounted for 29–66% of Mn_t_. It was higher in depth 1 than in depths 2 and 3 of the mid- and toe-slope profiles, while it was accumulated in depth 3 of the upper-slope profile. The P_ox_ content ranged between 90 and 324 mg kg^−1^ and accounted for 30–65% of P_t_. The upper soil profile contained a higher average value of P_ox_ (225.7 mg kg^−1^) than the mid- (188.7 mg kg^−1^) and toe slope (199.7 mg kg^−1^) profile. The highest value of P_ox_ content was found in depth 1 of the three profiles.

The PSC varied between 13 and 32 mmol kg^−1^ and was higher in depths 1 and 2 than in depth 3 in the three profiles, particularly in the toe-slope profile. The DPS ranged between 12 and 36% with high proportions in depth 1 of all profiles (Table 4). The DPS_profile_ was calculated to 20 and 25% for the upper- and mid-slope profile, respectively (data not shown). The DPS_profile_ for the toe-slope profile was estimated to 27 or 28% depending on whether the not investigated Sw soil horizon (12 cm) was assumed to be more similar to the M or Sd soil horizon, respectively.

### Sequential P fractionation

**Fig. 1:**
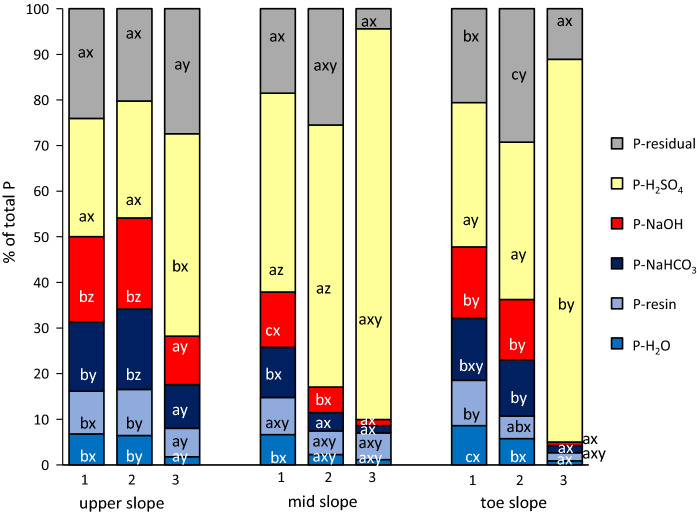
Mean proportions of P (% of total P) in different extracts of the sequential P fractionation for soils from three depths of the upper-, mid- and toe-slope soil profile. Different letters indicate significant differences in the proportion of one fraction between depths within the same soil profile (a–c) and between profiles within the same depth (x–z), respectively; *n* = 4

Highest proportions of P (44–86% of P_t_) were detected in the H_2_SO_4_ fraction (Fig. 1). In the upper- and toe-slope profiles, proportions of H_2_SO_4_ extractable P were significantly higher in depth 3 (44 and 84% of P_t_, respectively) compared with that in depths 1 or 2 of these profiles. In the mid-slope profile, significantly higher proportions of H_2_SO_4_ extractable P were detected in depths 1 and 2 (44 and 57% of P_t_) compared with the same depths of the other two profiles. NaOH-P accounted for 1 to 20% of P_t_, and its proportion was higher in depths 1 and 2 of each profile compared with that in depth 3 and decreased in the order: upper-slope > toe-slope > mid-slope profile. Proportions of labile P (H_2_O-P + resin-P + NaHCO_3_-P) ranged between 4 and 34% of P_t_. Labile P proportions decreased with increasing depth but were similar in depths 1 and 2 of the upper-slope profile. Residual P was similar within all profiles except within the toe-slope profile in which it was highest in depth 2 and lowest in depth 3.

### P *K*-edge XANES

The P *K*-edge XANES spectra of standards recorded at the CLS and TLS beamline, respectively, differed strongly in shape and intensity, while sample spectra were more similar (Fig. S1). Furthermore, for some standards (e.g., P adsorbed on goethite) it was not possible to acquire satisfying spectra at the TLS beamline. Due to the inconsistent variability and a reduced standard set for TLS, LCF resulted in non-comparable results. For this reason, we decided to continue only with spectra acquired at the CLS beamline.

**Fig. 2:**
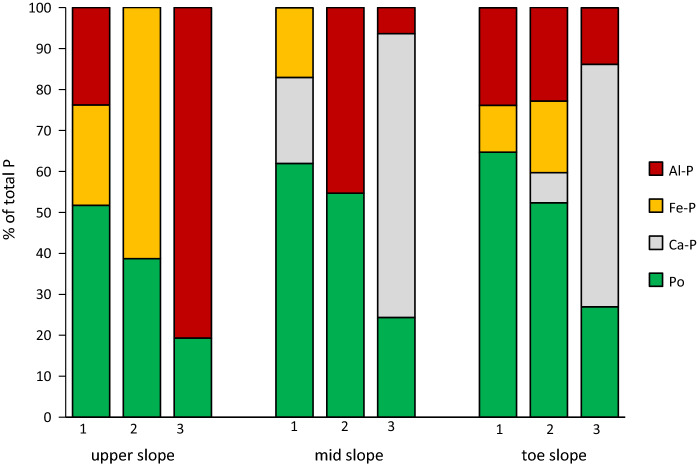
Proportions of P (% of total P) as detected by P *K*-edge XANES for soils from three depths of the upper-, mid- and toe-slope soil profile. Standards and spectra were recorded at the CLS-SXRMB beamline, Canada

Figure 2 represents the P species composition of soils as derived from LCF fits of spectra acquired at the CLS beamline (Fig. S2). Proportion of P_o_ species ranged between 19 and 65% of P_t_ and decreased with increasing soil depth at all three profiles. At mid-slope-3 and toe slope-3, Ca-P species represented 69% and 59% of P_t_, respectively. Fe-P and Al-P species accounted for 61 and 81% of P_t_ at upper slope-2 and 3, respectively. It should be noted that due to sample drying Fe-speciation may have been affected.

### P adsorption isotherms

**Fig. 3:**
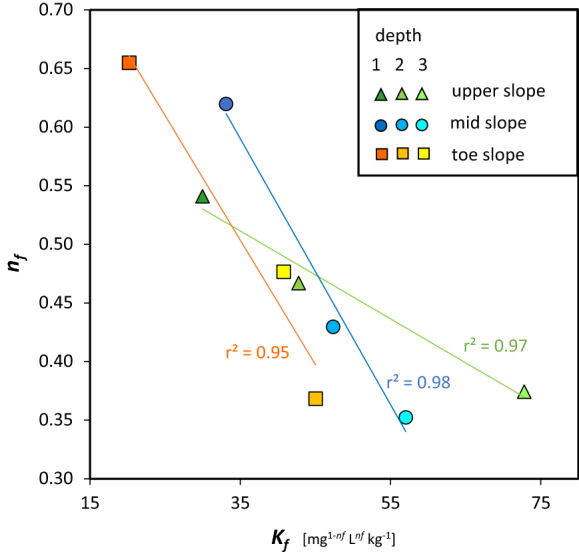
Mean *K*_f_ and *n*_f_ as derived from Freundlich isotherms for soils from three depths of the upper-, mid- and toe-slope soil profile. Parameters were calculated from the Freundlich equation $Q_{\text{ads}} \, = \,K_{\text{f}} \cdot C_{\text{eq}}^{{n_{\text{f}} }}$, where *Q*_ads_ is the mass of adsorbed P (mg kg^−1^), *C*_eq_ is the concentration of P in the equilibrated solution (mg L^−1^), *K*_f_ is the Freundlich unit capacity (${\text{mg}}^{{1 - n_{\text{f}} }} \,{\text{L}}^{{n_{\text{f}} }} \,{\text{kg}}^{ - 1}$) and *n*_f_ is the Freundlich exponent describing the nonlinearity of the adsorption; coefficient correlations of each profile were connected by linear regression lines and *r*^2^ is given in the plot, *n* = 3

P adsorption of different P concentrations resulted in isotherms which generally were in better agreement with the Freundlich isotherm model (*r*^2^ ranging between 0.94 and 0.97) than with the Langmuir isotherm model (*r*^2^ ranging between 0.84 and 0.99; Table S2, Fig. S3). Therefore, Freundlich isotherms were used to describe the P adsorption behavior in the present soils. The Freundlich isotherms were characterized by low *K*_f_ (20.2–33.1 ${\text{mg}}^{{1 - n_{\text{f}} }} \,{\text{L}}^{{n_{\text{f}} }} \,{\text{kg}}^{ - 1}$) and high *n*_f_ (0.54–0.65) coefficients for depth 1 of all profiles (Fig. 3). A wide range of *K*_f_ coefficients was detected for depth 3 of all profiles (40.8–72.8 ${\text{mg}}^{{1 - n_{\text{f}} }} \,{\text{L}}^{{n_{\text{f}} }} \,{\text{kg}}^{ - 1}$), while depth 2 soils differed mainly by their *n*_f_ (0.37–0.47). Within each profile, coefficient correlations of different depths were well established along linear regression lines (*r*^2^ = 0.95–0.98; Fig. 3). The *K*_f_ was significantly negatively correlated with C_org_ (*r* = − 0.76) and P_o_ determined by P *K*-edge XANES (*r* = − 0.72), while *n*_f_ was significantly positively correlated with C_org_ (*r* = 0.86), P_o_ determined by P *K*-edge XANES (*r* = 0.67), P_ox_ (*r* = 0.77), and P_t_ (*r* = 0.82).

## Discussion

### Potential availability and mobilization risk of P from the topsoil layer

Total P concentrations in depth 1 of the profiles (470-557 mg kg^−1^; Table 3) were in the range of P_t_ concentrations reported for Ap horizons of agriculturally used mineral soils in Mecklenburg-Western Pomerania, Germany (235–562 mg kg^−1^; Leinweber and Ahl 2013). The relatively lower P_t_ concentrations in depth 1 of the upper-slope profile (470 mg kg^−1^) as compared to the other two profiles (548 and 557 mg kg^−1^) may have resulted from erosive soil losses at the upper slope along with P relocation and subsequent accumulation of this P-rich soil material in the lower slope positions in the course of soil erosion/deposition. These redistributions of topsoil material at the field scale have recently been described as typical for agricultural soils from glacial till in the Baltic Sea region (Jandl et al. 2019). Since P_t_ was positively correlated with C_org_ (*r* = 0.84, *P* ≤ 0.001; data not shown), it can be assumed that most of the P_t_ in depth 1 was either associated with metal oxide–organic matter complexes (e.g., orthophosphate) (Yan et al. 2016), incorporated into the humic matrix (phytate) (Gerke 2015) or bound within the microbial community (e.g., phospholipids) (Quideau et al. 2016). A high proportion of P_o_ species (52–65% of P_t_) in depth 1 of all profiles was determined by P *K*-edge XANES supporting this assumption (Fig. 2). It implies a low P mobilization potential from all upper soil depths in case microbial P immobilization exceeds microbial P mineralization (Zhang et al. 2018). However, since microbial P immobilization strongly depends on the total C/P ratio, a low C/P ratio of < 28 in depth 1 of the profiles (usually about 60 in microbes) (Zhang et al. 2018) points to high P mineralization and thus leachable P in all soils of depth 1. Phosphorus fractionation data revealed that 26–32% of P_t_ was labile P (Fig. 1), which may be most easily prone to leaching (Rupp et al. 2018).

Besides a major proportion of P_o_ species, P *K*-edge XANES detected P bound to Al and Fe(hydr)oxides, indicating that pedogenic oxides also play an important role in P adsorption particularly in depth 1 of the upper- and toe-slope profile (49 and 35% of P_t_, respectively; Fig. 2). Since the NaOH extract from the sequential P fractionation is a good measure of P bound to pedogenic oxides (Hedley et al. 1982), similar proportions of P in the NaOH extract compared with the P *K*-edge XANES results would be expected if all pedogenicoxides were easily accessible by the NaOH solution. However, only 19 and 16% of P_t_, respectively, were recovered in the NaOH extract, suggesting that the remaining proportion of P (30 and 19% of P_t_, respectively) was occluded within aggregates formed by sesquioxides which were not chemically attacked by NaOH solution (Hedley et al. 1982). Phosphorus occlusion within sesquioxide aggregates as well as the detection of some parts of P bound as apatite (mid-slope-1) may imply a comparably lower risk of P mobilization also under reducing conditions. However, if the pH value temporarily decreases as can be observed under reducing conditions (Ponnamperuma 1972), also P from these speciation/formations could dissolve, thus also leading to an increased risk of P mobilization from mid-slope-1 at least for a short period of time.

Phosphorus sorption capacity calculated from the oxalate extract ranged between 27 and 32 mmol P kg^−1^, suggesting that only a maximum of 1.5–2.1 times more P could be adsorbed compared with the present amount of P (15–18 mmol P kg^−1^). Although oxalate extraction neglects some clay binding sites for P as well as binding sites offered by crystalline pedogenic oxides (Rennert 2019), PSC calculated from oxalate extraction is commonly used to estimate P sorption capacity of Northern European soils because an empirical linear relationship between the sorption maximum of P and the sum of Al_ox_ and Fe_ox_ was reported by Beek (1979) (Maguire et al. 2001).

A relatively low binding capacity for P was also demonstrated by low *K*_f_ coefficients (20.2–33.1 ${\text{mg}}^{{1 - n_{\text{f}} }} \,{\text{L}}^{{n_{\text{f}} }} \,{\text{kg}}^{ - 1}$, Fig. 3, Table S2) of the Freundlich P adsorption isotherms at depth 1 of all soils (Yan et al. 2016), which may suggest a high risk of P mobilization. This is in line with our implications from P_t_, labile P and pedogenic oxides on potential P availability and mobilization risk. The relatively high *n*_f_ coefficients (0.54–0.65) in this soil depth demonstrated a heterogeneous adsorption surface (Lu et al. 2014) which was probably due to many different P binding sites provided by SOM and mineral particles. This was supported by the coincidence of high contents of C_org_ and of pedogenic oxides in depth 1 of all profiles. Generally, isotherms of the three soil profiles showed better conformity to the Freundlich rather than Langmuir model which can be explained by the soils’ surface heterogeneity (Wang et al. 2013).

### Potential P availability and P mobilization risk from the subsoil layers

Total P concentrations in subsoil layers were generally lower than in the topsoil layers (Table 3) following the well-described decrease of nutrient contents with increasing soil depth (e.g., Jobbágy and Jackson 2001). Only in the upper-slope profile, similar P_t_ concentrations of depths 1 and 2 were detected which may be due to soil disturbance in the course of drainage installation. This assumption was supported by a similar P pool distribution for depths 1 and 2 of the upper-slope profile (Fig. 1). Therefore, for the upper-slope profile, a potential P mobilization risk based on P_t_ and labile P is also relatively high from depth 2. In contrast, soil depths 2 and 3 of the mid-slope and toe-slope profiles suggest a lower P mobilization risk not just because there was less P_t_ which potentially could be leached but in particular because the P in these depths consisted of a higher portion of more stable P than labile P (Fig. 1).

P *K*-edge XANES showed decreasing proportions of P_o_ species with increasing soil depth, which was well in line with decreasing C_org_ concentrations (Fig. 2, Table 3). On the other hand, proportions of Al-, Fe- and Ca-P species increased and even dominated at depth 3 which, however, was not always reflected by total element concentrations of Al, Fe and Ca. This suggests an either preferred P binding to a certain type of (hydr)oxide in soil and/or the predominant occurrence of these elements in compounds and minerals that are not involved in phosphate binding. Judging from P *K*-edge XANES results, a high potential P mobilization can be assumed for depth 2 mainly of the upper- and toe-slope profiles since there were high proportions of P bound to Fe (hydr)oxides. Under reducing conditions, they would most likely be prone to reductive dissolution and release P. In addition, in depth 2 of the upper-slope profile relatively high P proportions of NaOH extractable P were detected indicating Fe- and Al-bound P (Hedley et al. 1982), while in depth 2 of the other two profiles the stable P fraction (H_2_SO_4_ fraction) was largest. This may suggest that in the mid- and toe-slope profiles P was occluded in sesquioxides (e.g., micropores of sesquioxide aggregates) or was physically encapsulated (McGroddy et al. 2008) which could lead to a lower potential P availability and thus also lower P mobilization risk under reducing conditions in these profiles. In depth 3 of the upper-slope soil profile, P *K*-edge XANES revealed high proportions of Al-P, suggesting a higher mobilization risk compared with depth 3 of the other two profiles in which Ca-P dominated. Also, 11% of P_t_ was extractable by NaOH which could point to a fair P proportion to be more easily prone to mobilization under reducing conditions compared with depth 3 of the other profiles (1% of P_t_).

Mainly in depth 3 of the mid- and toe-slope profile, Ca-P occurred which was in line with very high proportions of stable P from sequential fractionation. In this profile position, abundance of Ca-P can be explained by the well-known calcite content of glacial till (about 33% of CaCO_3_, equivalent to about 50% calcite in size fractions < 6.3 µm; Leinweber and Reuter 1992) where its weathering may have resulted in Ca-leftovers which acted as binding partners for phosphate. Non- or less-weathered parts of glacial till containing primary apatite are likely to occur deeper in the soil profile, while Ca from liming would be more likely to occur in depth 1 but is very unlikely in depth 3. Since P within apatite is most likely more stable under reducing conditions compared with P sorbed to Al and Fe (hydr)oxides, the P mobilization risk from depth 3 of the mid- and toe-slope profiles is assumed to be relatively low.

According to the isotherms of depths 2 and 3, a higher binding capacity for P (high *K*_f_ coefficients) was provided by the subsoil depths compared with depth 1 of the profiles. This may be explained by a reduction of P mineral binding sites in depth 1 compared to lower depths of the profiles due to SOM covering these sites particularly at depth 1 (Chassé and Ohno 2016). A higher binding capacity for P by subsoil depths implies that mobilized P from depth 1 may still be sorbed by the depths below and potentially be recycled by deep-rooting plants. However, it should be noted that preferential flow, which is an important path for draining water in soil (Stone and Wilson 2006), may bridge this potential P recovery zone.

### Potential P leaching risk from the profiles

The three profiles along the slope showed redoximorphic characteristics (Sg, Sw/Sd horizons according to the German Soil Classification system KA5 (AG Boden 2005)) indicating the effect of different oxygen levels in these soils over time. This suggests that drainage closure could again result in periodical changes from oxic to reducing conditions in these soils. Such changes may not only have affected P pools in the past but would most probably also affect them in the future with consequences for potential P availability, mobilization and potential P leaching risk.

The weighed mean DPS_profile_ indicated an enhanced potential P leaching risk for the mid- and toe-slope profiles (25%, 27/28%, respectively), if a threshold of 25% for mineral soils was assumed (Breeuwsma et al. 1995; Paulter and Sims 2000). Only the DPS_profile_ of the upper-slope soil profile (20%) was below the threshold, suggesting not much fear of an increased risk for this profile. However, interpretation of the P parameters at different soil depths within the upper-slope profile (see “Discussion” subsections above) may point toward an increase in potential P leaching risk also for this profile under reducing conditions.

## Conclusions

The complementary approach of P methods used in the present study was successful in assessing the potential availability of P from different points of view. In particular, the P species derived from synchrotron-based P *K*-edge XANES gave valuable information also about P occurrence under potentially reducing conditions. Within one experiment, however, care has to be taken when comparing P *K*-edge XANES spectra acquired at different synchrotrons since different experimental setups and detection efficiencies of beamlines may lead to differences in spectra characteristics.

Based on the majority of P soil characteristics (P_t_ content, proportions of labile/moderately labile/stable P, P species, P adsorption isotherms) and their complex interactions, our study suggests that fluctuation in water level and the associated changes in redox potential could lead to an increased P availability/P leaching risk at the three soil profiles. In particular, the upper-slope profile could be prone to increased P release mainly because of higher proportions of labile P in depth 2 of this profile compared with the other two profiles. To verify this assumption, further investigations of the redox-induced mobilization and speciation of P under systematic changes of redox potential in disturbed and undisturbed samples of these soils are required at the micro- and mesoscales using biogeochemical microcosms and lysimeters, respectively. Studies on P adsorption under different redox conditions may help to further describe the P binding and mobilization in these soils and to ascertain their potential contribution to P losses at the field scale more precisely.

## Electronic supplementary material

Below is the link to the electronic supplementary material.

Supplemental Fig. S1 Selected standard spectra (A) as well as sample spectra (B) analyzed at the Canadian beamline (CLS-SXRMB; black) and the Taiwanese beamline (TLS-16A; red), respectively (PPTX 291 kb)

Supplemental Fig. S2 Linear combination fits of P *K*-edge XANES sample spectra for soils from three depths (1–3) of the upper-, mid- and toe-slope soil profile. Only the best fit is shown. All shown samples and fitting standards were analyzed at the CLS-SXRMB beamline, Canada (PPTX 1480 kb)

Supplemental Fig. S3P adsorption isotherms for soils from three depths (1–3) of the upper-, mid- and toe-slope soil profile. *K*_f_ and *n*_f_ coefficients can be derived from the equation ($y\, = \,K_{\text{f}} \cdot x^{{n_{\text{f}} }}$); bars indicate standard deviation, *n* = 3 (PPTX 60 kb)

Supplementary material 4 (DOCX 46 kb)
