# Supplementary material for: Speciation and sorption of phosphorus in agricultural soil profiles of redoximorphic character
Source: Environ Geochem Health. 2020 Apr 23;42(10):3231–46. doi: 10.1007/s10653-020-00561-y (PMC7518995; doi:10.1007/s10653-020-00561-y)
Supplement: Supplementary file 3 — P adsorption isotherms for soils from three depths (1–3) of the upper-, mid- and toe-slope soil profile. Kf and nf coefficients can be derived from the equation ($y\, = \,K_{\text{f}} \cdot x^{{n_{\text{f}} }}$y=Kf·xnf); bars indicate standard deviation, n = 3 (PPTX 60 kb) [file 10653_2020_561_MOESM3_ESM.pptx]

## Slide 1
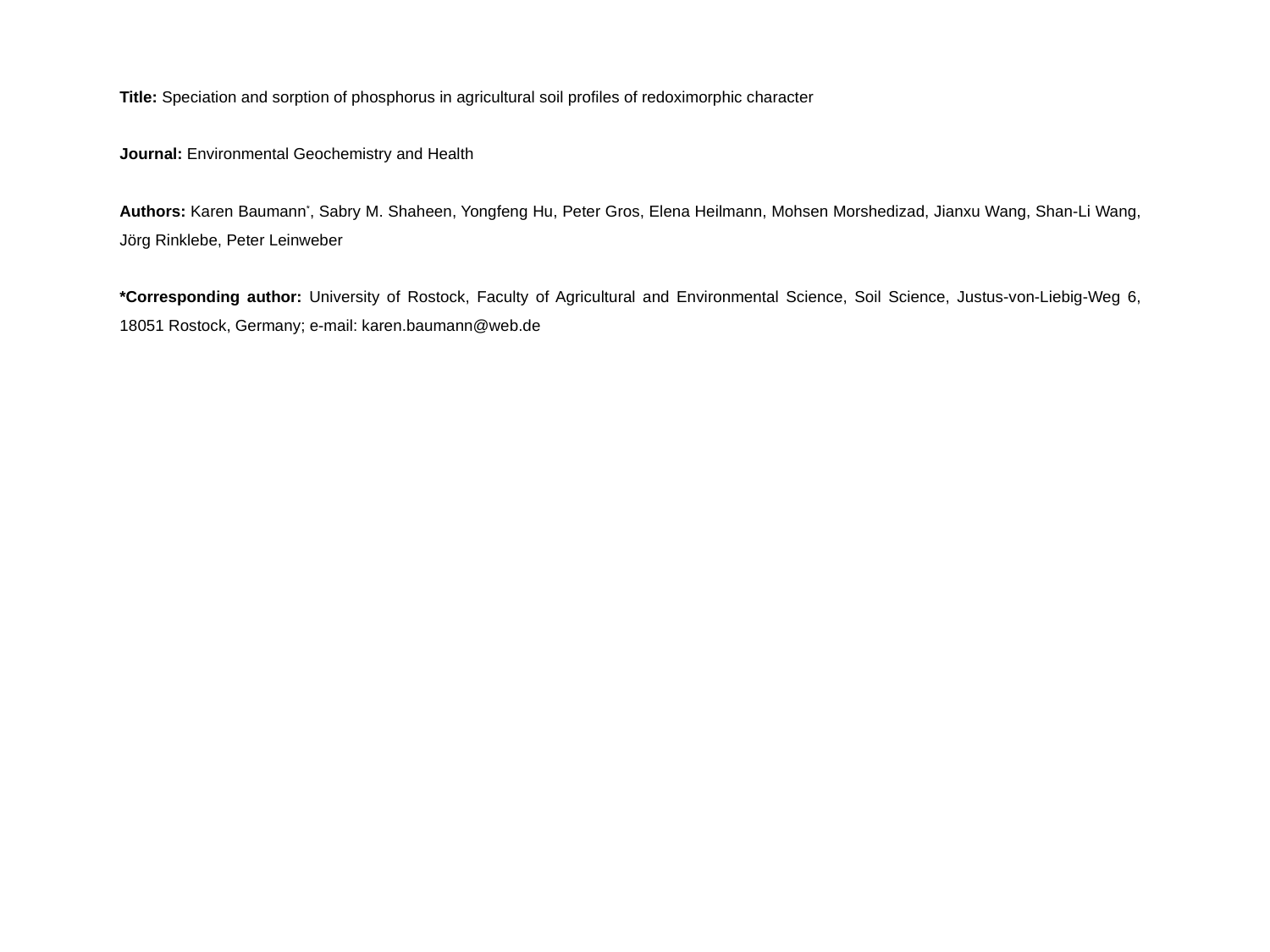

Title: Speciation and sorption of phosphorus in agricultural soil profiles of redoximorphic character
Journal: Environmental Geochemistry and Health
Authors: Karen Baumann*, Sabry M. Shaheen, Yongfeng Hu, Peter Gros, Elena Heilmann, Mohsen Morshedizad, Jianxu Wang, Shan-Li Wang, Jörg Rinklebe, Peter Leinweber
*Corresponding author: University of Rostock, Faculty of Agricultural and Environmental Science, Soil Science, Justus-von-Liebig-Weg 6, 18051 Rostock, Germany; e-mail: karen.baumann@web.de

## Slide 2
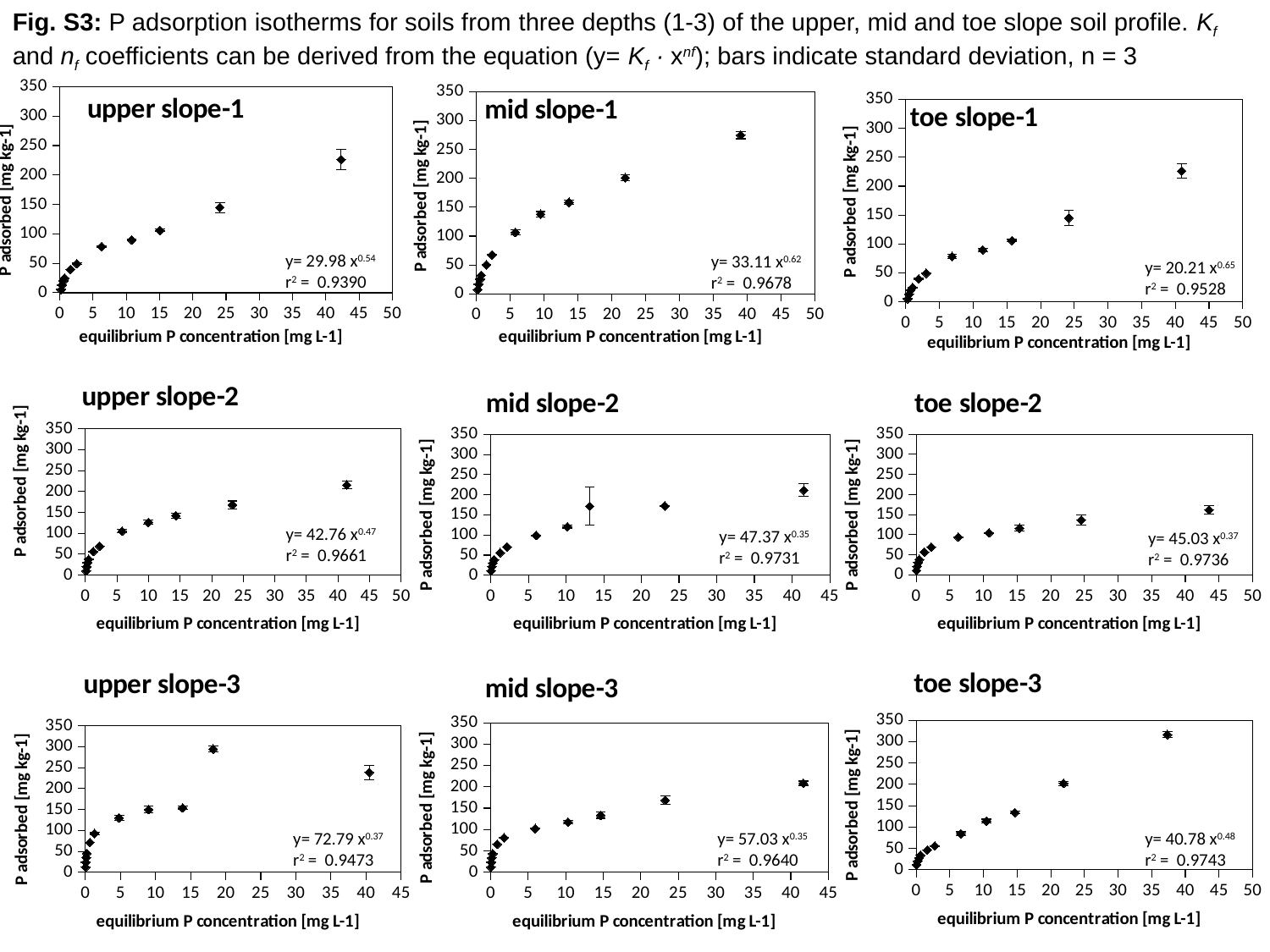

Fig. S3: P adsorption isotherms for soils from three depths (1-3) of the upper, mid and toe slope soil profile. Kf and nf coefficients can be derived from the equation (y= Kf ∙ xnf); bars indicate standard deviation, n = 3
### Chart: upper slope-1
| Category | |
|---|---|
### Chart: mid slope-1
| Category | |
|---|---|
### Chart: toe slope-1
| Category | |
|---|---|y= 29.98 x0.54
r2 = 0.9390
y= 33.11 x0.62
r2 = 0.9678
y= 20.21 x0.65
r2 = 0.9528
### Chart: upper slope-2
| Category | |
|---|---|
### Chart: toe slope-2
| Category | |
|---|---|
### Chart: mid slope-2
| Category | |
|---|---|y= 42.76 x0.47
r2 = 0.9661
y= 47.37 x0.35
r2 = 0.9731
y= 45.03 x0.37
r2 = 0.9736
### Chart: toe slope-3
| Category | |
|---|---|
### Chart: mid slope-3
| Category | |
|---|---|
### Chart: upper slope-3
| Category | |
|---|---|y= 72.79 x0.37
r2 = 0.9473
y= 40.78 x0.48
r2 = 0.9743
y= 57.03 x0.35
r2 = 0.9640
