# Supplementary material for: Speciation and sorption of phosphorus in agricultural soil profiles of redoximorphic character
Source: Environ Geochem Health. 2020 Apr 23;42(10):3231–46. doi: 10.1007/s10653-020-00561-y (PMC7518995; doi:10.1007/s10653-020-00561-y)
Supplement: Supplementary file 4 — Supplementary material 4 (DOCX 46 kb) [file 10653_2020_561_MOESM4_ESM.docx]

**Title:** Speciation and sorption of phosphorus in agricultural soil profiles of redoximorphic character

**Journal:** Environmental Geochemistry and Health

**Authors:** Karen Baumann*, Sabry M. Shaheen, Yongfeng Hu, Peter Gros, Elena Heilmann, Mohsen Morshedizad, Jianxu Wang, Shan-Li Wang,

Jörg Rinklebe, Peter Leinweber

***Corresponding author:** University of Rostock, Faculty of Agricultural and Environmental Science, Soil Science, Justus-von-Liebig-Weg 6,

18051 Rostock, Germany; e-mail: karen.baumann@web.de

**Table S1** Procedure and data acquisition parameters to gain highest possible spectra quality at the Canadian beamline (CLS-SXRMB) and at the Taiwanese beamline (TLS-16A)

|  | **CLS-SXRMB** | **TLS-16A** |
| --- | --- | --- |
| sample preparation | smeared as a thin film on double-sided P-free carbon tape | soil pellets were mounted in P-free carbon tape |
| recording mode | fluorescence | fluorescence |
| photon energy step size | 1 eV (2130 to 2140 eV), 0.15 eV (2140 to 2180 eV),  0.5 eV (2180 to 2200 eV) | 0.5 eV (2122 to 2140 eV), 0.15 eV (2140 to 2162 eV), 0.5 eV (2162 to 2200 eV) |
| dwell time reference compound | 1 s | 2 to 8 s |
| dwell time sample | 4 s | 2 to 8 s |
| number of spectra recorded | 2 to 3 | 2 to 3 |

**Table S2** Mean correlation coefficients of Freundlich (*K_f_*, *n_f_*) and Langmuir isotherms (*K_L_*, *q_L,max_*) for soils from three depths of the upper, mid and toe slope soil profile. n = 3

| hill position | depth | Freundlich | | | | | | | Langmuir | | | | | | |
| --- | --- | --- | --- | --- | --- | --- | --- | --- | --- | --- | --- | --- | --- | --- | --- |
|  |  | *K_f_* | | | *n_f_* | | | r² | *K_L_* | | | *q_L,max_* | | | r² |
|  |  | [mg^1-^*^nf^* L*^nf^* kg^-1^] | | |  | | |  | [L mg^-1^] | | | [mg kg^-1^] | | |  |
| upper slope | 1 | 29.98 | ± | 0.45 | 0.54 | ± | 0.02 | 0.9390 | 0.200 | ± | 0.0137 | 210.6 | ± | 135.8 | 0.8739 |
|  | 2 | 42.76 | ± | 0.38 | 0.47 | ± | 0.02 | 0.9661 | 0.714 | ± | 0.0142 | 145.9 | ± | 14.0 | 0.9879 |
|  | 3 | 72.79 | ± | 0.67 | 0.37 | ± | 0.02 | 0.9473 | 4.169 | ± | 0.1870 | 133.5 | ± | 20.9 | 0.9413 |
| mid slope | 1 | 33.11 | ± | 0.38 | 0.62 | ± | 0.02 | 0.9678 | 0.094 | ± | 0.0030 | 448.2 | ± | 292.5 | 0.9693 |
|  | 2 | 47.37 | ± | 0.35 | 0.43 | ± | 0.01 | 0.9731 | 2.467 | ± | 0.1283 | 90.7 | ± | 11.5 | 0.9226 |
|  | 3 | 57.03 | ± | 0.42 | 0.35 | ± | 0.01 | 0.9640 | 5.464 | ± | 0.3803 | 89.4 | ± | 13.4 | 0.8694 |
| toe slope | 1 | 20.21 | ± | 0.36 | 0.65 | ± | 0.03 | 0.9528 | 0.004 | ± | 0.0002 | 5163.3 | ± | 94207.3 | 0.9178 |
|  | 2 | 45.03 | ± | 0.29 | 0.37 | ± | 0.01 | 0.9736 | 3.011 | ± | 0.1132 | 84.4 | ± | 7.0 | 0.9580 |
|  | 3 | 40.78 | ± | 0.33 | 0.48 | ± | 0.01 | 0.9743 | 3.881 | ± | 0.3002 | 67.1 | ± | 8.7 | 0.8435 |
